# Prevalence and Early Left Ventricular Outcomes of Occlusive Myocardial Infarction in Non-ST-Elevation Acute Coronary Syndrome: A Retrospective Observational Study

**DOI:** 10.7759/cureus.113628

**Published:** 2026-07-29

**Authors:** Surender Himral, Jai Bharat Sharma, Shivali Sandal, Iva V Patel, Kunal Mahajan

**Affiliations:** 1 Cardiology, Himachal Heart Institute, Mandi, IND; 2 Critical Care Medicine, Himachal Heart Institute, Mandi, IND

**Keywords:** coronary angiography, ejection fraction, nstemi, occlusive myocardial infarction, timi flow

## Abstract

Background and objective: Acute coronary syndromes are classified by the electrocardiogram (ECG) into ST-elevation myocardial infarction (STEMI) and non-ST-elevation types. However, many patients with non-ST-elevation acute coronary syndrome (NSTE-ACS) have a fully blocked culprit artery, a condition called occlusive myocardial infarction (OMI). We aimed to find how common OMI is in an Indian NSTE-ACS cohort and to compare its early effects on heart function and survival with non-occlusive myocardial infarction (NOMI).

Methods: In this retrospective observational study, 340 patients presenting within 24 hours of chest pain with NSTE-ACS who underwent coronary angiography at a tertiary cardiac centre were enrolled. OMI was defined angiographically as a totally or near-totally occluded culprit artery (Thrombolysis in Myocardial Infarction (TIMI) flow grade 0-1) and NOMI as grade 2-3. Left ventricular ejection fraction (LVEF) was measured at baseline and at three months, and all-cause death was recorded at 9-12 months. Logistic regression identified factors independently associated with OMI.

Results: OMI was found in 112 of 340 patients (32.9%, 95% confidence interval (CI) 28.2-38.1%) and NOMI in 228 (67.1%). The two groups had similar baseline features and risk factors, but non-ST-elevation myocardial infarction (NSTEMI) was more common in the OMI group (83/112 (74.1%) vs. 140/228 (61.4%); p=0.028). OMI patients had a lower baseline LVEF (44.1 ± 11.4% vs. 49.2 ± 11.1%; p<0.001), which stayed lower at three months (50.4 ± 8.3% vs. 53.4 ± 8.5%; p=0.010) despite improvement in both groups. On regression, a lower baseline LVEF was the factor most strongly associated with OMI. Death rates were similar (1/88 (1.1%) vs. 6/175 (3.4%); p=0.49).

Conclusions: About one in three NSTE-ACS patients undergoing coronary angiography in this single-centre, retrospective cohort had a blocked culprit artery, and this group had worse heart function at presentation and at three months despite similar baseline risk profiles. Given the retrospective, single-centre design and incomplete follow-up, these findings are hypothesis-generating: they support maintaining a high index of suspicion for a blocked artery in NSTE-ACS patients with reduced LVEF, but do not establish that earlier angiography improves outcomes in this subgroup.

## Introduction

For decades, the 12-lead electrocardiogram (ECG) has served as the backbone of acute coronary syndrome triage, splitting patients into ST-elevation myocardial infarction (STEMI) and non-ST-elevation acute coronary syndrome (NSTE-ACS) categories. Emergency care pathways still hinge on this split, since it decides who goes straight for reperfusion. STEMI typically signals a sudden, complete blockage of a coronary artery, so restoring flow without delay is critical. NSTE-ACS, on the other hand, is a broader and more variable category encompassing both non-ST-elevation myocardial infarction (NSTEMI) and unstable angina in which the ECG by itself often fails to capture what is actually happening inside the coronary artery [1,2].

A growing body of work has revealed that many patients initially labelled as NSTEMI in fact turn out, on angiography, to have a totally occluded culprit vessel. This entity, termed occlusive myocardial infarction (OMI), appears to affect close to a third of NSTEMI patients, yet they are typically routed through conventional NSTEMI protocols rather than fast-tracked for immediate reperfusion [2]. Because occlusions of the inferior, posterior, or lateral walls, especially those involving the left circumflex artery, often generate only faint or no ST-segment changes on a standard ECG, the underlying occlusion can easily go unnoticed. This diagnostic blind spot can delay revascularisation even while the myocardium continues to suffer ischaemic injury [3,4].

Rather than relying on ECG morphology, the OMI framework reorients attention toward whether an artery is acutely occluded, highlighting a group of patients who stand to gain from earlier reperfusion. Reported OMI prevalence, however, differs considerably between populations, and evidence specific to South Asian cohorts is still sparse. Establishing how common OMI is locally and what it means clinically matters for judging whether existing triage and diagnostic pathways are catching the patients who need urgent attention.

With this gap in mind, we designed the present study to estimate how frequently OMI occurs among consecutive Indian patients presenting with NSTE-ACS and to compare its short-term effects on cardiac function and clinical outcomes against non-occlusive myocardial infarction (NOMI).

## Materials and methods

Study design and population

This was a retrospective observational study. We enrolled consecutive patients who came within 24 hours of chest pain with NSTE-ACS and who had coronary angiography at our tertiary cardiac centre. NSTE-ACS was diagnosed using the Fourth Universal Definition of Myocardial Infarction [5]. Patients who had previous bypass surgery and those who had STEMI at the same time were excluded. The study was approved by the institutional ethics committee, and all patients gave written informed consent before enrolment.

During the study period, patients presenting with suspected NSTE-ACS were referred for coronary angiography according to our institution's standard angiography referral protocol for NSTE-ACS. Patients were not excluded from angiography on the basis of Thrombolysis in Myocardial Infarction (TIMI) risk score [6], troponin level, or ECG findings alone.

Angiographic assessment and group definition

Every patient had coronary angiography, and flow in the culprit artery was graded using the TIMI scale [6]. We defined OMI as TIMI flow grade 0-1 (no or very little forward flow) and NOMI as TIMI grade 2-3. The culprit artery was chosen based on how it looked on angiography together with the ECG and wall-motion findings. We deliberately used a strict angiographic definition of OMI based on a totally or near-totally occluded culprit artery (TIMI 0-1). This differs from the broader electrocardiographic OMI paradigm, which additionally captures subtotal occlusion (TIMI 2) and spontaneously reperfused occlusion (TIMI 3 with a high peak troponin); our estimate should therefore be read as the prevalence of a totally occluded culprit artery and represents a conservative lower bound for OMI. The time from symptom onset to coronary angiography was recorded for every patient.

Culprit vessel and TIMI flow grade were assigned by two independent interventional cardiologists, blinded to each other's assessment and to clinical and echocardiographic data, with discrepancies resolved by consensus discussion.

Echocardiographic and outcome assessment

Left ventricular ejection fraction (LVEF) was measured by transthoracic echocardiography within 48 hours of admission and again at the three-month follow-up. For the categorical analysis, we grouped LVEF as normal (≥50%), mildly reduced (40-49%), or moderately to severely reduced (<40%). All-cause death was checked at 9-12 months of follow-up.

LVEF was calculated using the biplane Simpson's method. All echocardiograms were independently read by two blinded readers, who were unaware of the patient's angiographic OMI/NOMI status and TIMI flow grade at the time of image interpretation, with discrepancies resolved by consensus.

Statistical analysis

Continuous variables are given as mean ± standard deviation and were compared between groups with the independent-samples t-test. The change in LVEF from baseline to three months within each group was tested with the paired t-test, and three-month LVEF was compared between groups using analysis of covariance (ANCOVA) adjusted for baseline LVEF to account for regression to the mean. Categorical variables are given as counts and percentages and were compared with the chi-squared or Fisher’s exact test, whichever fitted. We used logistic regression to identify factors independently associated with OMI, reporting adjusted odds ratios (aORs) with 95% confidence intervals (CIs); with 112 OMI events and seven candidate covariates, the events-per-variable ratio (approximately 16) was adequate for stable estimation. The prevalence of OMI is reported with a 95% CI. All analyses used the available data for each variable (complete-case analysis), and the number of patients contributing to each comparison is reported. A two-sided p-value below 0.05 was taken as significant. All statistical analyses were performed using IBM SPSS Statistics, version 26 (IBM Corp., Armonk, USA).

This study was designed as a descriptive, prevalence-focused observational study rather than a comparative study powered around a pre-specified primary contrast; consequently, no formal a priori sample size calculation was performed. The achieved sample of 340 patients yielded a 95% CI of 28.2-38.1% around the primary prevalence estimate of 32.9%, indicating adequate precision for the study's descriptive aim. For the multivariable logistic regression, the events-per-variable ratio of approximately 16 (112 OMI events across seven candidate covariates) exceeded conventional thresholds for stable model estimation.

Of the 340 patients in the analysis cohort, baseline LVEF was available in 336 (four missing data/not retrieved), three-month LVEF was available in 298 (42 missing data/not retrieved), and mortality status at 9-12 months was available in 263 (77 missing data/not retrieved).

## Results

**Figure 1:**
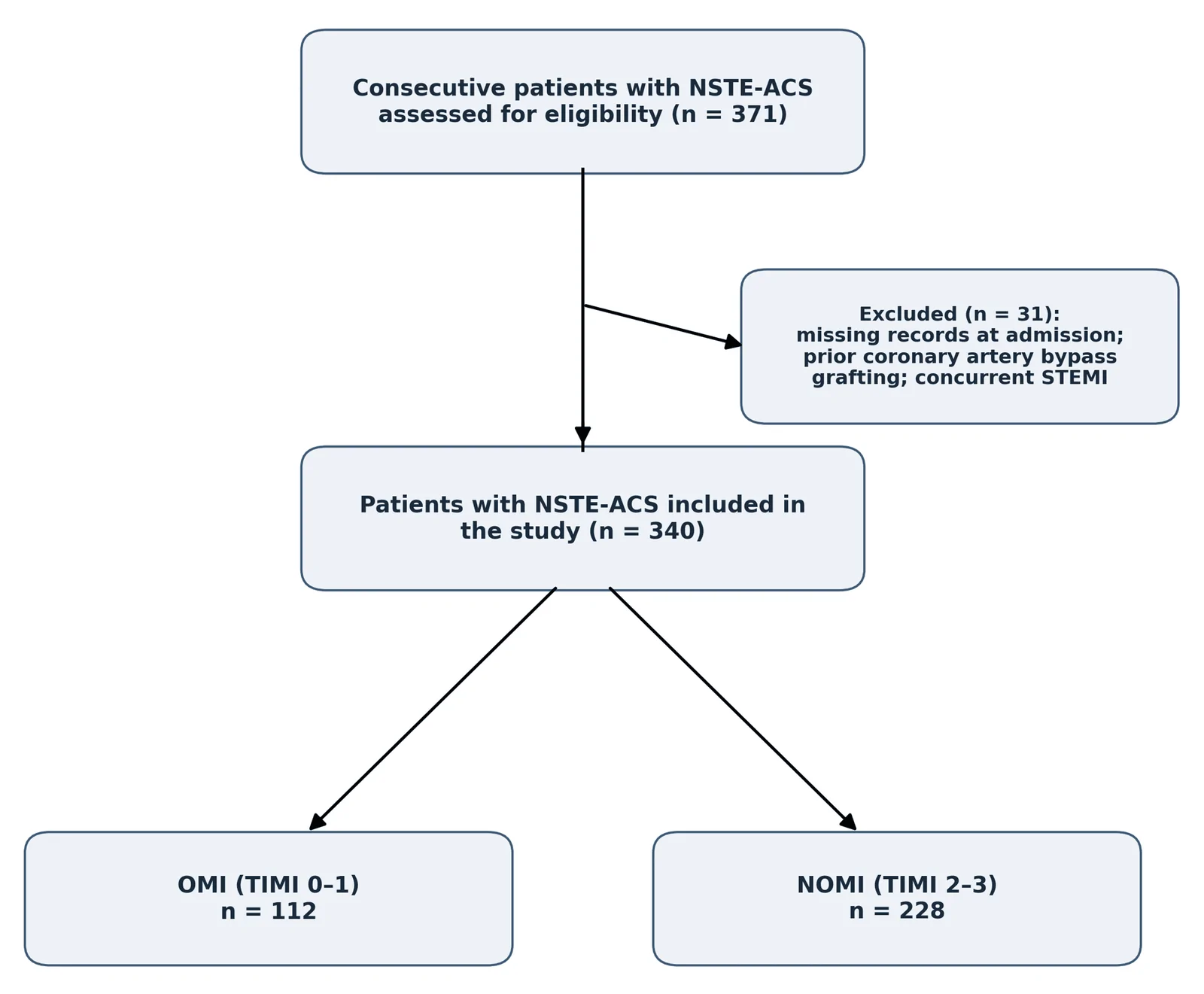
Participant flow through the study OMI: Occlusive myocardial infarction; NOMI: Non-occlusive myocardial infarction; TIMI: Thrombolysis in Myocardial Infarction; LVEF: Left ventricular ejection fraction; CABG: Coronary artery bypass grafting; STEMI: ST-elevation myocardial infarction; NSTE-ACS: Non-ST-elevation acute coronary syndrome

Of the total 371 consecutive patients with NSTE-ACS assessed for eligibility during the study period, 31 patients were excluded (prior coronary artery bypass grafting; concurrent STEMI), leaving 340 patients who underwent coronary angiography and formed the analysis cohort (Figure 1). 

A visual summary of the primary study parameters and key findings is presented in Appendix A.

Cohort characteristics and OMI prevalence

The study included 340 patients with a mean age of 64.2 ± 11.3 years, and 253 (74.4%) were men. The most common comorbidity was hypertension (205, 60.3%), followed by diabetes (126, 37.1%). NSTEMI made up 223 of 340 cases (65.6%), and the rest were unstable angina. On angiography, 112 patients (32.9%, 95% CI 28.2-38.1%) had OMI and 228 (67.1%) had NOMI. Their baseline characteristics are shown in Table 1.

**Table 1:** Baseline clinical characteristics of the study cohort *p<0.05. OMI: Occlusive myocardial infarction; NOMI: Non-occlusive myocardial infarction; NSTEMI: Non-ST-elevation myocardial infarction; SD: Standard deviation

Variable	Total (n=340)	OMI (n=112)	NOMI (n=228)	p-value
Age (years), mean ± SD	64.2 ± 11.3	63.3 ± 12.0	64.7 ± 10.9	0.28
Male sex, n (%)	253 (74.4)	77 (68.8)	176 (77.2)	0.12
Hypertension, n (%)	205 (60.3)	69 (61.6)	136 (59.6)	0.82
Diabetes mellitus, n (%)	126 (37.1)	37 (33.0)	89 (39.0)	0.34
Current smoker, n (%)	93 (27.4)	27 (24.1)	66 (28.9)	0.45
Prior statin use, n (%)	95 (27.9)	25 (22.3)	70 (30.7)	0.14
NSTEMI presentation, n (%)	223 (65.6)	83 (74.1)	140 (61.4)	0.028^*^

As shown in Table 1, the two groups were largely similar at baseline. There were no significant differences in age, sex, hypertension, diabetes, smoking, or prior statin use. The one clear difference was the type of presentation: NSTEMI was more common in OMI patients than in NOMI patients (83/112 (74.1%) vs. 140/228 (61.4%); p=0.028).

The distribution of TIMI flow grades reflected the predefined classification of the study groups and is presented in Table 2. Among the 112 patients in the OMI group, 80 (71.4%) had TIMI grade 0 flow and 32 (28.6%) had TIMI grade 1 flow, with no patients demonstrating TIMI grade 2 or 3 flow. Conversely, among the 228 patients in the NOMI group, 24 (10.5%) had TIMI grade 2 flow and 204 (89.5%) had TIMI grade 3 flow, while none had TIMI grade 0 or 1 flow. Overall, in the study population of 340 patients, TIMI grade 0, 1, 2, and 3 flow were observed in 80 (23.5%), 32 (9.4%), 24 (7.1%), and 204 (60.0%) patients, respectively.

**Table 2:** Distribution of culprit-artery TIMI flow grade by group OMI was defined as TIMI grade 0-1 and NOMI as TIMI grade 2-3. TIMI: Thrombolysis in Myocardial Infarction; OMI: Occlusive myocardial infarction; NOMI: Non-occlusive myocardial infarction

TIMI flow grade	OMI (n=112)	NOMI (n=228)	Total (n=340)
Grade 0, n	80 (71.4%)	0	80 (23.5%)
Grade 1, n	32 (28.6%)	0	32 (9.4%)
Grade 2, n	0	24 (10.5%)	24 (7.1%)
Grade 3, n	0	204 (89.5%)	204 (60.0%)

The left anterior descending artery was the most common culprit vessel in the NOMI group (140/228, 61.4%) compared with the OMI group (45/112, 40.2%), and this difference was statistically significant (p=0.0003) (Table 3). Conversely, left circumflex artery involvement was significantly more frequent in the OMI group than in the NOMI group (33/112 (29.5%) vs. 34/228 (14.9%), p=0.003). Right coronary artery involvement was comparable between the groups (33/112 (29.5%) vs. 52/228 (22.8%), p=0.127), while Ramus artery involvement was rare and similar in both groups (1/112 (0.9%) vs. 2/228 (0.9%), p=1.000).

**Table 3:** Distribution of culprit coronary arteries in OMI and NOMI groups Data are presented as n (%). Percentages are column percentages within each group. The p-values are derived from the chi-squared test, with Fisher's exact test used for the Ramus intermedius owing to small expected cell counts. The overall distribution of culprit vessels differed significantly between groups (χ²=17.4, df=3, p<0.001). LAD: Left anterior descending artery; LCx: Left circumflex artery; RCA: Right coronary artery; OMI: Occlusive myocardial infarction; NOMI: Non-occlusive myocardial infarction; df: Degrees of freedom

Culprit vessel	OMI (n=112)	NOMI (n=228)	p-value
LAD	45 (40.2%)	140 (61.4%)	0.0003
LCx	33 (29.5%)	34 (14.9%)	0.003
RCA	33 (29.5%)	52 (22.8%)	0.127
Ramus	1 (0.9%)	2 (0.9%)	1.000

Left ventricular function

Differences in heart function were clear right from presentation (Table 4). Baseline LVEF was lower in the OMI group than in the NOMI group (44.1 ± 11.4% vs. 49.2 ± 11.1%; p<0.001). When grouped into categories, fewer OMI patients had a normal LVEF (30.6% vs. 53.3%) and more had moderate-to-severe dysfunction (26.1% vs. 16.0%); the overall category distribution differed significantly between groups (p<0.001).

**Table 4:** Left ventricular function and clinical outcomes: OMI versus NOMI *p<0.05. The p-value for each LVEF category block is the overall test across all three categories at that timepoint. Percentages within each category block are calculated from patients with available echocardiographic data. The mortality denominator reflects patients with available follow-up data. LVEF: Left ventricular ejection fraction; pp: Percentage points; SD: Standard deviation; CI: Confidence interval; OMI: Occlusive myocardial infarction; NOMI: Non-occlusive myocardial infarction; ANCOVA: Analysis of covariance

Outcome	OMI (n=112)	NOMI (n=228)	p-value
LVEF at baseline (%), mean ± SD	44.1 ± 11.4	49.2 ± 11.1	<0.001^*^
LVEF at 3 months (%), mean ± SD	50.4 ± 8.3	53.4 ± 8.5	0.010^*^
Change in LVEF (baseline to 3 months)	+6.3 pp	+4.2 pp	0.18
Adjusted follow-up LVEF difference (ANCOVA)	-0.4 pp (95% CI -1.7 to +0.9 )	Reference	0.58
LVEF category at baseline, n (%)	(n=111)	(n=225)	
Normal (≥50%)	34 (30.6)	120 (53.3)	<0.001^*^
Mildly reduced (40-49%)	48 (43.2)	69 (30.7)	
Moderate-to-severe (<40%)	29 (26.1)	36 (16.0)	
LVEF category at 3 months, n (%)	(n=100)	(n=198)	
Normal (≥50%)	43 (43.0)	127 (64.1)	0.002^*^
Mildly reduced (40-49%)	44 (44.0)	54 (27.3)	
Moderate-to-severe (<40%)	13 (13.0)	17 (8.6)	
All-cause mortality, n/N (%)	1/88 (1.1)	6/175 (3.4)	0.49

At three months, LVEF improved within both groups (paired p<0.001 for each), but OMI patients still had a lower ejection fraction than NOMI patients (50.4 ± 8.3% vs. 53.4 ± 8.5%; p=0.010). The share of patients with a normal LVEF rose in both groups but stayed lower in the OMI arm (43.0% vs. 64.1%), and moderate-to-severe dysfunction fell in both groups (13.0% vs. 8.6%); again, the overall distribution differed (p=0.002). The crude LVEF gain was numerically larger in OMI patients (+6.3 vs. +4.2 percentage points; p=0.18); however, because the OMI group started from a lower baseline, this unadjusted comparison is susceptible to regression to the mean. After adjustment for baseline LVEF (ANCOVA), three-month LVEF remained lower in the OMI group (adjusted mean difference -0.4 pp; 95% CI -1.7 to +0.9; p=0.58), indicating that the presenting deficit was not fully recovered over this period.

Factors independently associated with OMI

Factors independently associated with OMI were assessed by multivariable logistic regression, including age, sex, hypertension, diabetes, current smoking, type of presentation, and baseline LVEF. The results are shown in Table 5.

**Table 5:** Multivariable logistic regression: factors independently associated with OMI *p<0.05. Model based on 336 patients with complete data. OR: Odds ratio; CI: Confidence interval; LVEF: Left ventricular ejection fraction; NSTEMI: Non-ST-elevation myocardial infarction

Variable	Adjusted OR (95% CI)	p-value
Baseline LVEF (per 1% increase)	0.97 (0.94-0.99)	0.002^*^
Age (per 1 year)	0.97 (0.95-1.00)	0.03^*^
Male sex	0.62 (0.35-1.08)	0.09
NSTEMI presentation	1.49 (0.86-2.56)	0.15
Diabetes mellitus	0.69 (0.41-1.13)	0.14
Hypertension	1.21 (0.72-2.02)	0.47
Current smoking	0.72 (0.40-1.30)	0.27

Baseline LVEF was the variable most strongly associated with OMI. The aOR was 0.97 for every 1% rise in LVEF (95% CI 0.94-0.99; p=0.002). Because the odds ratio is below 1, a higher ejection fraction lowered the odds of OMI, which means that the lower the LVEF, the higher the chance of a blocked artery. Because baseline LVEF and culprit-artery flow were assessed during the same admission, this relationship reflects a cross-sectional association - a low presenting LVEF accompanying, rather than preceding, an occluded artery - and should be read as a bedside marker that raises suspicion rather than as a temporal predictor. Older age was weakly and inversely associated with OMI (aOR 0.97 per year, 95% CI 0.95-1.00; p=0.03), while sex, hypertension, diabetes, smoking, and NSTEMI presentation were not independently associated after adjustment. The modest inverse association with age should be regarded as hypothesis-generating; it may reflect a more thrombotic, plaque-rupture phenotype in younger patients rather than the diffuse, collateralised disease more typical of older patients.

Mortality

Follow-up death data were available for 263 patients (88 OMI and 175 NOMI). One OMI patient (1.1%) and six NOMI patients (3.4%) died, a difference that was not significant (p=0.49). With so few deaths, we could not draw firm conclusions about the difference in death risk between the groups.

## Discussion

This retrospective analysis of 340 patients with NSTE-ACS found OMI in 112 individuals, or 32.9% of the cohort. An NSTEMI presentation was more frequent among those with OMI than among those with NOMI (74.1% vs. 61.4%; p=0.028), while the two groups were otherwise comparable in baseline profile and cardiovascular risk burden. Patients with OMI did, however, present with a markedly lower LVEF, and although both groups showed improvement by three months, the OMI group's LVEF remained the lower of the two. Mortality overall was low and did not differ meaningfully between groups - a finding best explained by the short observation window, the small number of deaths recorded, and the fact that every patient in the cohort underwent invasive management.

The frequency of OMI we recorded sits comfortably within the range reported elsewhere. Koyama and colleagues documented coronary occlusion or flow-limiting disease in as many as 63% of suspected NSTEMI patients sent for immediate angiography, a figure inflated relative to ours largely because their definition also captured flow-limiting stenoses alongside complete blockages [3]. By contrast, the meta-analysis conducted by Hung et al., pooling data from 25 studies and nearly 61,000 NSTEMI patients, arrived at a prevalence of 34% - remarkably close to our own 32.9% [2]. Comparable figures have emerged from Soon et al. (24%) and Sharma et al. (32.5%) [7,8]. Taken together, this body of evidence points to a sizeable subset of patients carrying an NSTEMI label who, in reality, have an acutely blocked artery that the initial ECG fails to flag.

Complete coronary occlusion also left its mark on cardiac performance in our cohort. Baseline LVEF was significantly lower among OMI patients than among their NOMI counterparts (44.1 ± 11.4% vs. 49.2 ± 11.1%; p<0.001), echoing Hung et al.'s observation that an occluded culprit vessel independently tracks with reduced LVEF and elevated peak biomarker levels [2]. Wei and colleagues reported a similar pattern of depressed LVEF in NSTEMI patients with total occlusion of the culprit artery [9]. In our own multivariable model, a low baseline LVEF stood out as the single strongest independent correlate of OMI, implying that depressed ventricular function at presentation ought to raise clinical suspicion of an occluded artery even when the ECG shows no diagnostic ST-segment elevation.

The clinical picture we observed also lines up with earlier work. In a national registry spanning more than 131,000 patients, Terlecki et al. characterised totally occluded NSTEMI as an intermediate-risk category subject to longer treatment delays, largely because left circumflex occlusions rarely produce the classic ECG signature of STEMI [10]. Our own data mirror this: NSTEMI presentations were significantly more common in the OMI group, and angiography confirmed that left circumflex involvement was likewise more frequent in OMI than in NOMI (29.5% vs. 14.9%; p=0.003). Right coronary artery involvement trended higher in the OMI group as well, though this difference did not reach statistical significance (29.5% vs. 22.8%; p=0.127). Given that circumflex occlusion so often escapes detection on a standard 12-lead ECG, its over-representation among OMI patients offers a plausible reason why these cases slip through initial evaluation.

These observations carry practical weight for how NSTE-ACS patients are assessed. Given the poor sensitivity of a standard 12-lead ECG for posterior and lateral wall infarction - particularly when the left circumflex artery is the culprit - supplementary diagnostic approaches could sharpen early detection. In a cohort of 219 NSTEMI patients studied before urgent angiography, Horie et al. used a synthesised 18-lead ECG and identified circumflex occlusion with TIMI 0-1 flow in 26.5% of cases, a figure close to the 29.5% we observed [11]. Their work further showed that posterior ST-segment elevation on synthesised leads V7-V9 independently predicted both circumflex occlusion and more extensive myocardial damage. Read alongside our results, these findings make a case for incorporating posterior leads, repeated ECGs, and serial high-sensitivity troponin testing when patients have ongoing symptoms or reduced LVEF despite a nondiagnostic initial ECG.

A recurring concern in the literature is that patients with OMI who fall outside conventional STEMI criteria often face substantial treatment delays. Meyers et al. found that STEMI-negative OMI patients waited a median of 437 minutes for coronary angiography, versus just 41 minutes for their STEMI-positive counterparts, despite comparably poor outcomes [12]. In a related vein, Herrera et al. found that among 665 cancelled STEMI activations, 28 patients (4.2%) were nevertheless found to have an acutely occluded artery, identified only through subtle ECG changes [13]. Both observations reinforce our own findings and underline that leaning solely on ST-segment elevation risks missing a meaningful number of patients with a fully occluded artery.

Other studies have linked OMI to higher rates of death and heart failure, yet we found no significant mortality gap between our two groups [2,7]. The short duration of follow-up, the handful of deaths recorded, and the fact that both groups received invasive treatment are the most plausible explanations for this. Even so, the persistently lower LVEF among OMI patients points to a substantial burden of myocardial damage from complete occlusion, regardless of whether short-term survival looks similar between groups.

Limitations

This study has several limitations. It was conducted at a single centre, which may limit the generalizability of the findings. The prevalence of OMI was estimated only among patients who underwent coronary angiography and therefore may not reflect the true prevalence in all patients presenting with NSTE-ACS. TIMI flow was assessed only at the time of angiography, and spontaneous recanalisation before the procedure may have resulted in some patients with prior occlusion being classified as NOMI. Furthermore, the strict definition of OMI based on TIMI 0-1 flow may underestimate the true burden of clinically relevant coronary occlusion.

Follow-up data were incomplete, with baseline LVEF available in 336 patients, three-month LVEF in 298 patients, and mortality status in 263 of the 340 enrolled patients. Loss to follow-up may therefore have influenced the assessment of ventricular recovery and clinical outcomes. Because angiographic findings and LVEF were measured during the same hospitalisation, the association between the two should be interpreted as cross-sectional rather than causal. Finally, the small number of deaths and the relatively short follow-up period limited our ability to detect differences in mortality or evaluate longer-term outcomes such as recurrent myocardial infarction, heart failure, or ventricular remodelling. Larger multicentre studies with longer follow-up are needed to better define the long-term clinical significance of OMI in patients presenting with NSTE-ACS.

Taken together, the retrospective, single-centre, angiography-selected design of this study, combined with incomplete follow-up, means that the associations reported here, particularly between baseline LVEF and OMI status, should be regarded as hypothesis-generating rather than confirmatory, and cannot support causal or predictive claims, nor conclusions about the benefit of any specific management strategy such as earlier angiography.

## Conclusions

In this single-centre, retrospective cohort of NSTE-ACS patients undergoing coronary angiography, about one in three had a totally or near-totally occluded culprit artery (OMI), and this group had lower LVEF both at presentation and at three months, despite broadly similar baseline risk profiles. A lower baseline LVEF was independently associated with OMI on multivariable analysis, though this relationship was assessed cross-sectionally and should be interpreted as a bedside marker that raises clinical suspicion rather than as a validated predictor. Short-term mortality did not differ significantly between groups, but this comparison involved few events and incomplete follow-up and should not be read as evidence of equivalent risk. Given the retrospective, single-centre, angiography-selected design, these findings are hypothesis-generating: they suggest that a meaningful proportion of NSTE-ACS patients may harbour an occluded culprit artery not flagged by ECG, and that reduced LVEF at presentation may be a useful bedside cue for this possibility. Whether earlier angiography or altered management improves outcomes in this subgroup cannot be answered by this design and should be tested in larger, multicentre, prospective studies with longer and more complete follow-up.

## Figures and Tables

**Figure 2:**
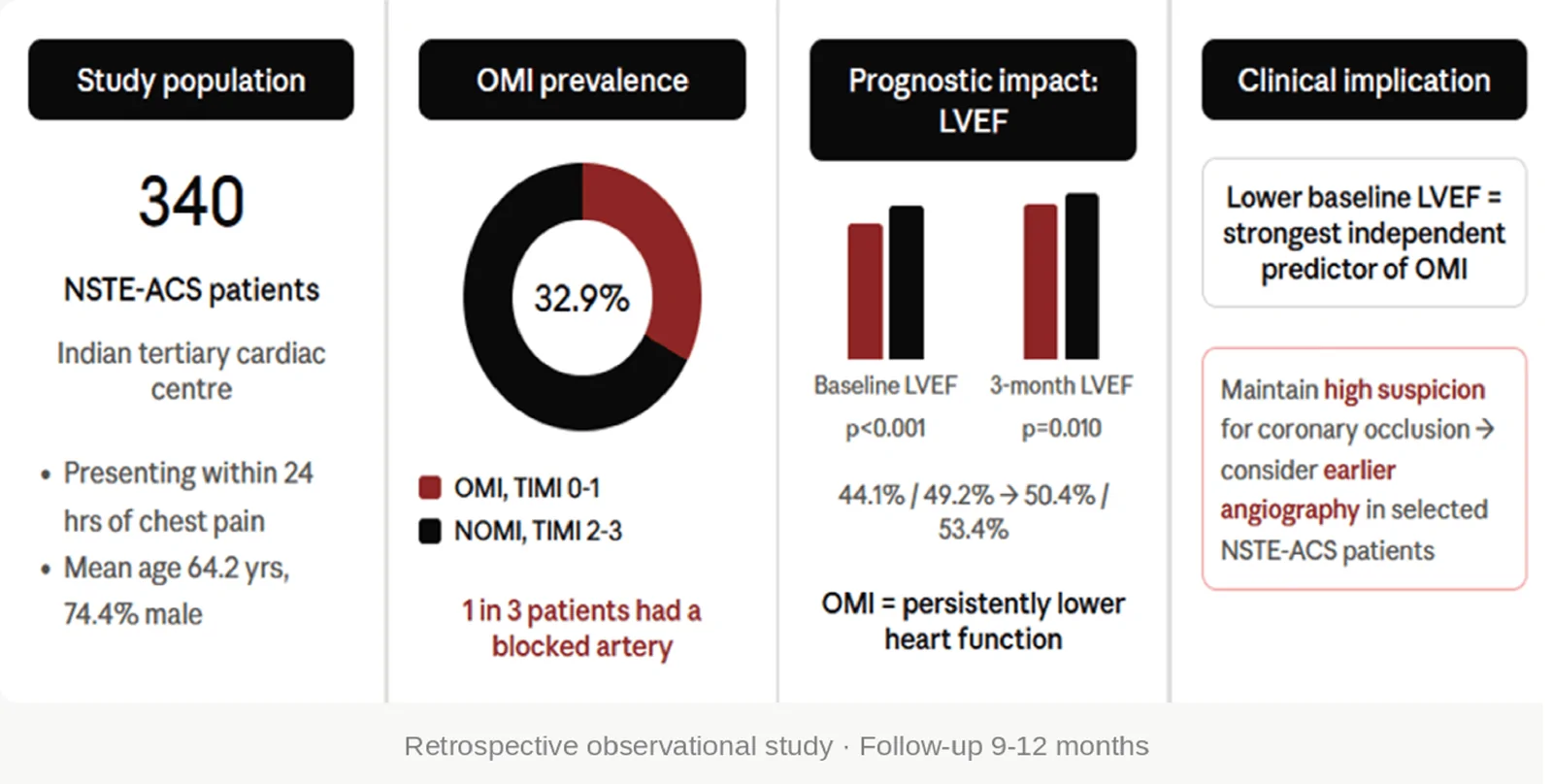
Graphical abstract The figure was created using Microsoft PowerPoint (Microsoft Corp., Redmond, USA). NSTE-ACS: Non-ST-elevation acute coronary syndrome; OMI: Occlusive myocardial infarction; NOMI: Non-occlusive myocardial infarction; TIMI: Thrombolysis in Myocardial Infarction; LVEF: Left ventricular ejection fraction

## Appendices

Appendix A 
